# The effects of low-carbohydrate diet on glucose and lipid metabolism in overweight or obese patients with T2DM: a meta-analysis of randomized controlled trials

**DOI:** 10.3389/fnut.2024.1516086

**Published:** 2025-01-06

**Authors:** Wende Tian, Shuyu Cao, Yongxin Guan, Zihao Zhang, Qiyu Liu, Jianqing Ju, Ruixi Xi, Ruina Bai

**Affiliations:** ^1^National Clinical Research Center for Chinese Medicine Cardiology, Xiyuan Hospital, China Academy of Chinese Medical Sciences, Beijing, China; ^2^Changping Traditional Chinese Medicine Hospital, Beijing, China; ^3^Liaoning University of Chinese Medicine, Shenyang, China

**Keywords:** low-carbohydrate diet, glucose and lipid metabolism, type 2 diabetes mellitus, overweight, obesity

## Abstract

**Background:**

The dual burden of Type 2 Diabetes Mellitus (T2DM) and obesity is a critical public health issue. Low-carbohydrate diets have emerged as a potential intervention, yet clinical evidence remains inconclusive.

**Purpose:**

This meta-analysis assesses the impact of low-carbohydrate diets on metabolic profiles in overweight or obese T2DM patients, aiming to guide clinical practice.

**Methods:**

A systematic review identified randomized clinical trials (RCTs) comparing low-carbohydrate diets to control diets in T2DM patients from PubMed, Embase, and the Cochrane Library databases up to April 2023.

**Results:**

Seventeen RCTs, encompassing 1,197 participants, demonstrated that low-carbohydrate diets significantly improved HbA1c levels and fasting plasma glucose (mean difference [MD] = −0.36, 95% CI −0.44 to −0.29, *p* < 0.00001; MD = −10.71, 95% CI −14.39 to −7.03, *p* < 0.00001). They also reduced triglycerides and increased HDL cholesterol (MD = −19.91, 95% CI −28.83 to −10.99, *p* < 0.00001; MD = 2.49, 95% CI 1.07–3.91, *p* = 0.0006), without affecting LDL and total cholesterol. Weight loss, reduced BMI, lower diastolic blood pressure, and decreased waist circumference were additional benefits.

**Conclusion:**

Low-carbohydrate diets may enhance glycemic control and lipid profiles in overweight or obese T2DM patients, warranting consideration in T2DM management. However, the variability in diet definitions and methodologies underscores the necessity for further research to standardize dietary guidelines and evaluate long-term effects.

## 1 Introduction

The increasing global incidence of Type 2 Diabetes Mellitus (T2DM), coupled with growing obesity rates, demands novel strategies for dietary management (1). The intertwining of T2DM with obesity not only complicates therapeutic strategies but also heightens the risk of associated metabolic and cardiovascular complications (2–4). As a result, there is an urgent demand for adjunct therapies that can help achieve metabolic goals. Among these, dietary interventions have emerged as one of the most promising approaches, as evidenced by the growing body of research in this area (5, 6). Low-carbohydrate diets are associated with the production of ketone bodies due to increased fatty acid oxidation and the upregulation of ketogenic enzymes (7). These diets also contribute to appetite suppression (8), improved postprandial glucose metabolism (9), and a reduction in insulin-like growth factor 1, which may influence metabolic health (10). Increasing evidence suggests that low-carbohydrate diets may offer benefits in managing several pathologies, such as diabetes, neoplasms, gastrointestinal and lung diseases (9), diseases of the cardiovascular system (11), as well as obesity (12, 13). In this context, low-carbohydrate diets have attracted significant attention for their potential in managing T2DM, based on the premise that reducing carbohydrate intake can mitigate hyperglycemia and promote weight loss.

Although all low-carbohydrate regimens aim to limit carbohydrate intake, there is no clear consensus on the precise definition of a “low-carbohydrate diet.” According to the American Diabetes Association, a low-carbohydrate diet is one that provides fewer than 130 g of carbohydrates per day, or less than 26% of total energy intake from carbohydrates (14, 15). Within this framework, low-carbohydrate diets are further categorized into moderate (<45–40% of total energy intake), low (<40–30%), and very low (<30–33%) carbohydrate diets. Typically, a low-carbohydrate diet is characterized by being low in digestible carbohydrates, high in fat, and moderate in protein (16–18). Over time, several variations of these diets have emerged, including the Atkins diet, modified Atkins diet, ketogenic diet, modified ketogenic diet, very low-calorie ketogenic diet, and ketogenic Mediterranean diet. These versions generally allow a higher carbohydrate intake (30–50 g per day) compared to others (19). However, confusion often arises regarding the distinction between low-carbohydrate high-fat diets and ketogenic diets, as the terms are sometimes used interchangeably. Despite these variations, the underlying principle of all low-carbohydrate diets remains the same: to reduce carbohydrate intake and promote fat as the body’s primary energy source.

Several systematic reviews and meta-analyses, particularly in Europe and the United States, have examined the effects of carbohydrate restriction in individuals with T2DM. These studies consistently report significant reductions in Hemoglobin A1c (HbA1c) levels within 6–12 months in carbohydrate-restricted groups compared to controls (including those following regular, high-carbohydrate, and low-fat diets) (20–24). However, these benefits tend to diminish after 12–24 months. Additionally, reduced carbohydrate intake, along with lower insulin doses, has been associated with a decrease in body weight in adults with T2DM (25–29). The actual carbohydrate content of the diet, however, may influence the observed benefits. For instance, moderately low or low-carbohydrate diets may be effective for weight reduction, while very low-carbohydrate diets may not be ideal for diabetic patients (30). In fact, such restrictive diets may adversely affect lipid profiles in this subgroup (31). Interestingly, a recent randomized clinical trial comparing very low-carbohydrate diets with low-carbohydrate diets in obese Japanese individuals with metabolic disorders found that both diets led to similar weight loss outcomes. Participants in both groups adhered to their respective diets and reported high satisfaction over a short-term period of 2 months (32).

Despite these promising results, the debate surrounding the long-term efficacy and safety of low-carbohydrate diets persists. Concerns about potential nutritional deficiencies and their impact on renal function have been raised (33), with studies such as those by Dehghan et al. (34) highlighting that the sustainability and long-term health outcomes—particularly in populations adhering to extreme dietary patterns—require further investigation. Moreover, the impact of low-carbohydrate diets on overall health, particularly in relation to dietary fiber intake, is a critical issue. A reduction in fiber can have serious consequences for gut health and overall well-being (35).

The American Diabetes Association has recently recognized low-carbohydrate eating patterns as an effective approach for managing diabetes. However, the absence of such diets in the Dietary Guidelines for Americans raises concerns about the flexibility of dietary recommendations to accommodate individual metabolic needs (36, 37). Furthermore, the lack of uniformity in the definition of “low-carbohydrate” diets across studies complicates the interpretation of the literature and the application of findings to clinical practice. This variability extends to differences in study populations, intervention durations, and measured outcomes, which hinder the ability to draw definitive conclusions (38, 39).

To our knowledge, no meta-analyses have specifically investigated the effects of low-carbohydrate diets on overweight or obese patients with T2DM. Addressing this gap, our meta-analysis aims to provide a clearer understanding of how low-carbohydrate diets can help manage T2DM, particularly in overweight and obese individuals. By focusing on randomized controlled trials with strict inclusion criteria, we aim to reduce variability and enhance the applicability of our findings. Ultimately, our goal is to strengthen the evidence for dietary interventions in T2DM management and inform clinical guidelines that consider the individual needs and preferences of patients.

## 2 Methods

### 2.1 Protocol and guidance

This systematic review and meta-analysis followed the PRISMA guidelines to ensure thorough and transparent reporting. We registered a predefined protocol in the PROSPERO database (registration number: CRD42024475967, https://www.crd.york.ac.uk/prospero/display_record.php?ID=CRD42024475967) to guarantee transparency and reproducibility in our methods.

### 2.2 Eligibility criteria

We selected randomized controlled trials (RCTs) that evaluated how low-carbohydrate diets affect glucose and lipid metabolism in overweight or obese adults with T2DM. Inclusion criteria were: (1) participants aged 18 or older with a T2DM diagnosis, (2) low-carbohydrate diets compared to any other diet, (3) outcomes measuring changes in HbA1c, total cholesterol (TC), low-density lipoprotein cholesterol (LDL-C), high-density lipoprotein cholesterol (HDL-C), and triglycerides (TG), and (4) a minimum intervention duration of 8 weeks. We excluded non-randomized studies, abstracts, conference presentations, and studies lacking relevant metabolic outcomes.

### 2.3 Information sources and search strategy

A comprehensive literature search was performed using PubMed, EMBASE, and the Cochrane Central Register of Controlled Trials databases from their inception until April 2023. We developed a search strategy using a combination of keywords and medical subject headings (MeSH) related to “low-carbohydrate diet, “Very-low-carbohydrate ketogenic diet,” “low carbohydrate high-protein diet,” “Type 2 Diabetes Mellitus,” “obesity,” “overweight,” “glucose metabolism,” and “lipid metabolism.” No language restrictions were imposed. The reference lists of identified reviews and included studies were also scanned for additional relevant studies.

### 2.4 Study selection

Two reviewers independently screened the titles and abstracts of all identified records to determine their eligibility. For studies that appeared potentially relevant, the full-text articles were thoroughly reviewed. Any disagreements between the reviewers were resolved through discussion, or if needed, by consulting a third reviewer.

### 2.5 Data collection

Two independent reviewers used a standardized form to extract data from each study included. This data encompassed study details (authors, publication year, country), participant demographics (age, sex, initial weight, BMI), specifics of the intervention (diet type, duration), comparator information, and relevant outcomes (changes in HbA1c, TC, LDL-C, HDL-C, and TG).

### 2.6 Risk of bias assessment

To ensure the integrity and validity of the included studies, we rigorously employed the Cochrane Collaboration’s tool for assessing the risk of bias in randomized trials. This detailed evaluation addressed several pivotal domains: the generation of random sequences, the concealment of allocation, the blinding of both participants and personnel, the blinding of outcome assessments, the completeness of outcome data, the selectivity of reporting, and the identification of any additional biases. To uphold accuracy and objectivity, we systematically resolved any discrepancies among reviewers either through consensus or, when necessary, by arbitration from a third, impartial reviewer.

### 2.7 Quality of evidence

We evaluated the quality of evidence for each outcome using the Grading of Recommendations, Assessment, Development, and Evaluations (GRADE) approach. This robust framework assesses evidence quality across several critical domains: study limitations, result inconsistencies, indirectness of evidence, imprecision, and potential publication bias. Following this methodical evaluation, the quality of evidence was classified into four distinct categories: high, moderate, low, or very low.

### 2.8 Data synthesis and analysis

To synthesize the data, we utilized Review Manager (RevMan version 5.4, Nordic Cochrane Center, Cochrane Collaboration) and R software (version 4.0.3; R Foundation for Statistical Computing). Employing random-effects models, we calculated pooled mean differences (MDs) or standardized mean differences (SMDs) for continuous outcomes, each accompanied by 95% confidence intervals (CIs), to account for the anticipated heterogeneity among studies. This variability was measured using the I^2^ statistic, with values exceeding 50% indicating significant heterogeneity. We also planned subgroup analyses considering factors such as the duration of the intervention, initial HbA1c levels, and the extent of carbohydrate restriction. Additionally, sensitivity analyses were performed to examine the impact of individual studies on the overall results and to ensure the robustness of our conclusions.

### 2.9 Additional analyses

When sufficient data were available, we conducted subgroup analyses to investigate potential sources of heterogeneity, including age, gender, BMI, and duration of diabetes. These analyses aimed to discern how these factors might influence outcomes across different studies. Additionally, sensitivity analyses were planned to evaluate the impact of study quality on our findings. This involved excluding studies deemed to have a high risk of bias to ensure that our results remained robust and reliable.

## 3 Results

### 3.1 Study selection and characteristics

**Figure 1:**
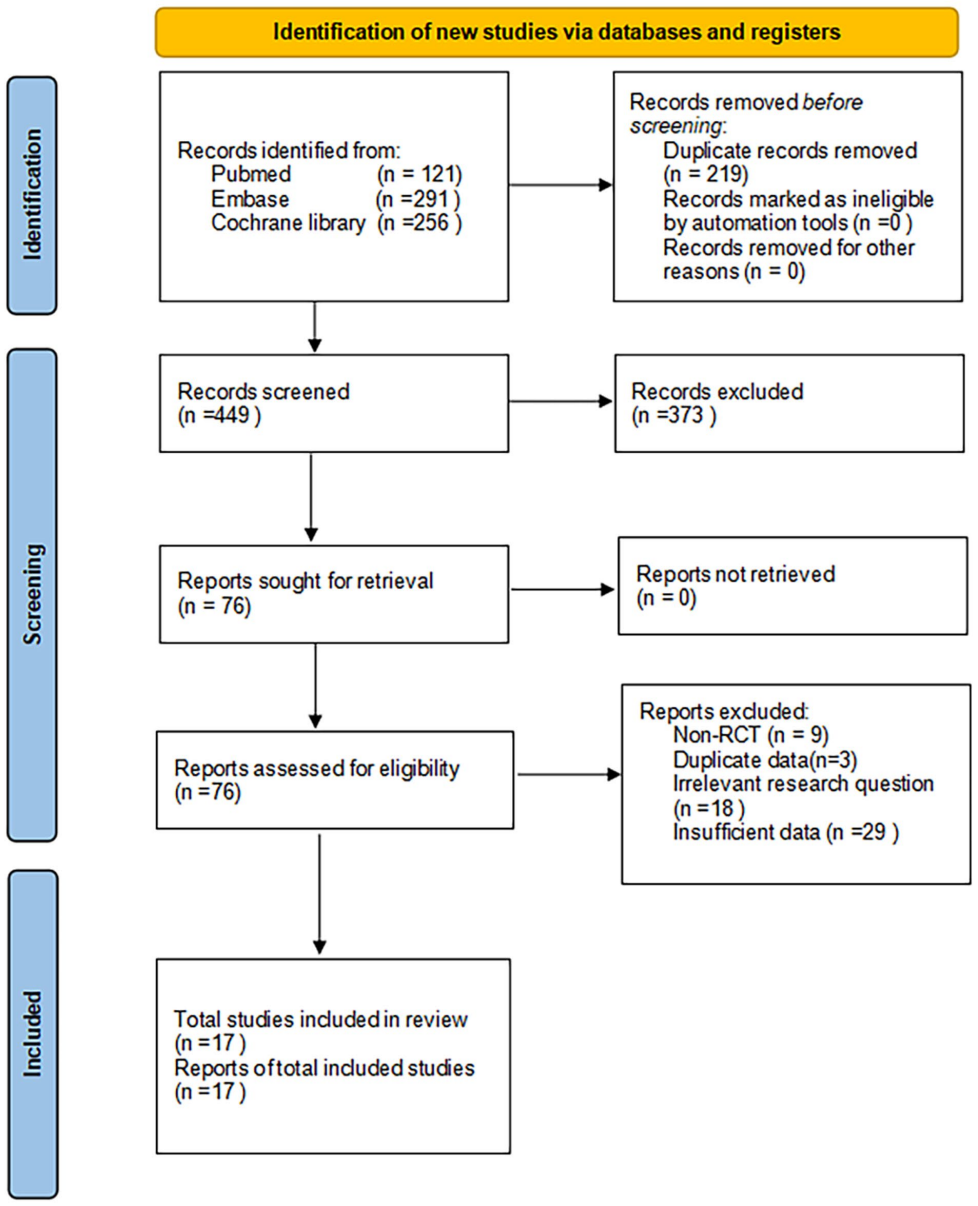
Flow diagram of the study selection progress.

The PRISMA flow diagram (Figure 1) offers a systematic overview of the trial selection process. Our extensive search yielded 668 articles. Using NoteExpress software, we excluded 219 duplicate publications, leaving 449 articles for further scrutiny. A subsequent review of titles and abstracts led to the elimination of 373 articles, narrowing our focus to 76 for detailed analysis. Upon full-text examination, we discarded 59 articles for various reasons, such as non-randomized controlled trial (RCT) designs, duplicate data, inadequate outcome measures, or irrelevant research questions. This rigorous screening process resulted in the inclusion of 17 studies, encompassing 1,197 participants.

These selected studies met our predefined inclusion criteria, targeting adults with overweight/obesity and T2DM who were undergoing low-carbohydrate diets compared to various control diets. The cohort comprised 643 individuals in the experimental groups and 554 in the control groups, including both male and female participants. Geographically, five studies were conducted in the United States, one in Australia, one in Canada, two each in Denmark and Switzerland, and one each in Germany, Spain, China, and Japan. The dietary interventions in the experimental groups involved restricting carbohydrate intake to below 26%.

Of the 17 studies, 15 assessed blood glucose levels, 15 evaluated blood lipid profiles, and 14 analyzed body weight changes. Table 1 provides detailed characteristics of the exposures and outcomes for each trial, facilitating a comprehensive comparison across studies.

**Table 1:** Characteristics of the 17 randomized controlled trials.

Trails	Study period	Country	Population	Gender	Experience/Control				Duration of intervention (months)
					Sample size	Diet	Age	BMI (kg/m^2^)	
Dorans, K S (2022) (40)	2018.9–2021.6	USA	T2DM	Both	73/69	LCD^1^/UD	59.3 ± 7.0/58.6 ± 8.8	36.6 ± 7.2/35.3 ± 6.0	6
Durrer, C (2021) (41)	2017.7.7–2019.4.1	Canada	T2DM	Both	78/60	Pharm-TCRD/TAUD	58 ± 11/59 ± 8	36.0 ± 6/35.1 ± 5.3	3
Hansen CD (2022) (42)	2016.1–2020.6	Denmark	T2DM	Both	92/42	LCHFD/HCLFD	57 ± 9/55 ± 12	33 ± 7/35 ± 8	6
Saslow, LR 1 (2017) (31)	Not specified	USA	T2DM/PreD	Both	14/15	VLCKD^1^/MCCRD^1^	≥18/ ≥18	>25/ >25	12
Saslow, LR 2 (2017) (43)	Not specified	USA	T2DM	Both	8/11	VLCKD^1^/LFD(CYPD)	53.0 ± 10.2/58.2 ± 6.7	≥25/ ≥25	8
Gram-Kampmann, EM (2022) (44)	2016.11–2018.12	Denmark	T2DM	Both	44/20	LCD^1^/HCFD	57.3 ± 0.9/55.2 ± 2.7	32.5 ± 0.9/35.2 ± 1.4	6
Chen, CY (2020) (45)	2017.6.15–2018.1.4	China	T2DM	Both	43/42	LCD^1^/TDD	63.1 ± 10.5/64.1 ± 7.4	27.31 ± 4.53/26.55 ± 3.69	18
Barbosa-Yañez, RL (2018) (46)	2013.10–2016.2	Germany	T2DM	Both	16/20	HVLCD/HLFD	63 ± 8/63 ± 8	32.1 ± 4.5/32.7 ± 4.9	3/4
Sato, J (2017) (46)	2013.9–2014.11	Japan	T2DM	Both	30/32	LCD^1^/CRD	60.5 ± 10.5/58.4 ± 10	27.27 ± 3.89/27.11 ± 4.27	6
Goday, A (2016) (47)	Not specified	Spain	T2DM	Both	45/40	VLCKD^2^/LCD^2^	54.89 ± 8.81/54.17 ± 7.97	33.3 ± 1.5/32.9 ± 1.6	4
Yamada, Y (2014) (48)	2011.4.1–2012.1.31	Japan	T2DM	Both	12/12	LCD^1^/CRD	63.3 ± 13.5/ 63.2 ± 10.2	24.5 ± 4.3/27.0 ± 3	6
Tay, J (2014) (49)	2012.5–2013.2	Australia	T2DM	Both	46/47	VLCFD/HCLFD	58 ± 7/ 58 ± 7	34.2 ± 4.5/35.1 ± 4.1	6
Jonasson, L (2014) (50)	Not specified	Sweden	T2DM	Both	29/30	LCD^1^/LFD	61 ± 9.5/63 ± 11	32 ± 5.1/34 ± 5.7	6
Guldbrand, H (2012) (51)	2008.8–2009.3	Sweden	T2DM	Both	18/17	LCD^1^/LFD	61.2 ± 9.5/62.7 ± 11	31.0 ± 4.5/31.6 ± 5	24
Davis, N J (2009) (52)	2004.8–2006.11	USA	T2DM	Both	55/50	LCD^1^/LFD	54 ± 6/ 53 ± 7	35 ± 6/37 ± 6	12
Saslow, L R 3 (2014) (53)	2012.9–2012.11	USA	T2DM/PreD	Both	16/18	VLCKD/MCCRD^2^	64.8 ± 7.7/55.1 ± 13.5	36.2 ± 8.2/37.4 ± 6.4	3
Li, S (2022) (54)	2018.6–2020.6	China	T2DM	Both	24/29	KD/TDD	36.5 ± 13.67/37.1 ± 14.02	29.04 ± 5.81/29.75 ± 6.07	3

LCD^1^, Low-carbohydrate diet; UD, Usual diet; Pharm-TCRD, Pharmacist-led therapeutic carbohydrate restriction diet; TAUD, guideline-based treatment-as-usual diet; LCHFD, low-carbohydrate, high-fat diet; HCLFD, high-carbohydrate, low-fat diet; VLCKD^1^, very low-carbohydrate ketogenic diet; MCCRD^1^, moderate-carbohydrate, calorie-restricted, low-fat diet; CYPD, American Diabetes Associations’ “Creat Your Plate” diet; HCFD, high carbohydrates and fiber diet; TDD, Traditional diabetes diet; HVLCD, hypocaloric very low carbohydrate diet; HCLFD, hypocaloric low fat diet; CRD, Calorie restricted diet; VLCKD^2^, Very low-calorie-ketogenic diets; LCD^2^, low-calorie diet; VLCFD, very-low-carbohydrate, high–unsaturated fat, low–saturated fat diet; HCLFD, high-Unrefined carbohydrate, low-fat diet; LFD, low-fat diet; MCCRD^2^, Medium carbohydrate, low fat, calorie-restricted, carbohydrate counting diet; KD, ketogenic diet.

### 3.2 Risk of bias assessment

The risk of bias in our reviewed studies was meticulously evaluated using the Cochrane Collaboration’s tool. Our analysis revealed that 52.94% of the studies demonstrated a low risk of bias concerning random sequence generation and allocation concealment. However, we observed significant challenges in blinding participants and personnel, a common issue in dietary intervention studies. Specifically, 12 studies detailed explicit randomization methods, and nine studies clearly described their allocation concealment processes.

Blinding of participants posed substantial difficulties, as 10 studies failed to blind participants to their respective interventions. Only five studies successfully implemented blinding for outcome assessors, while four reported incomplete data, potentially introducing biases in the results. Additionally, nine studies were free from other forms of bias. The intrinsic nature of dietary interventions often complicates achieving full blinding, leading to possible performance and detection biases.

**Figure 2:**
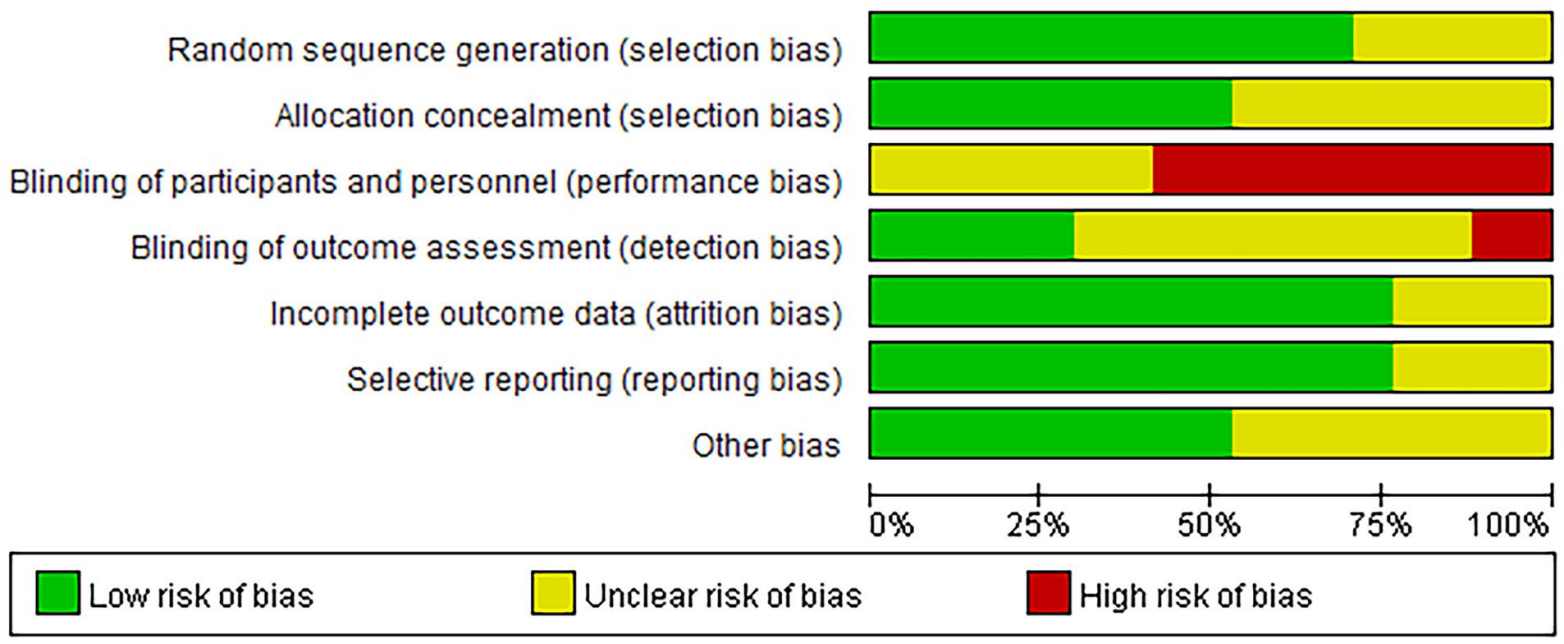
Risk of bias graph.

For a detailed visual representation of these bias assessments, please refer to Figure 2.

### 3.3 Main outcomes

#### 3.3.1 Effect of a carbohydrate diet on HbA1c and FPG

**Figure 3:**
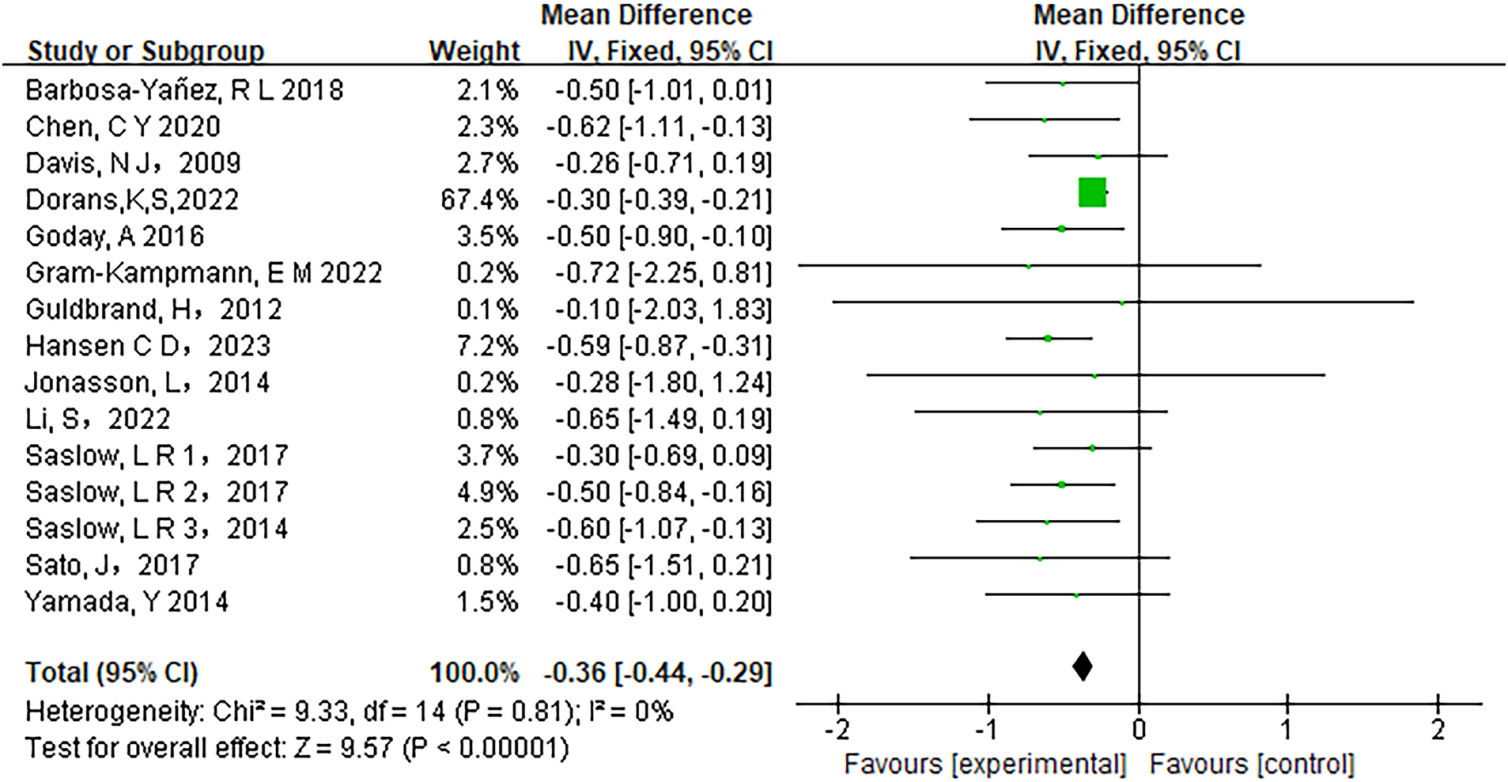
Effect of a carbohydrate diet on HbA1c.

**Figure 4:**
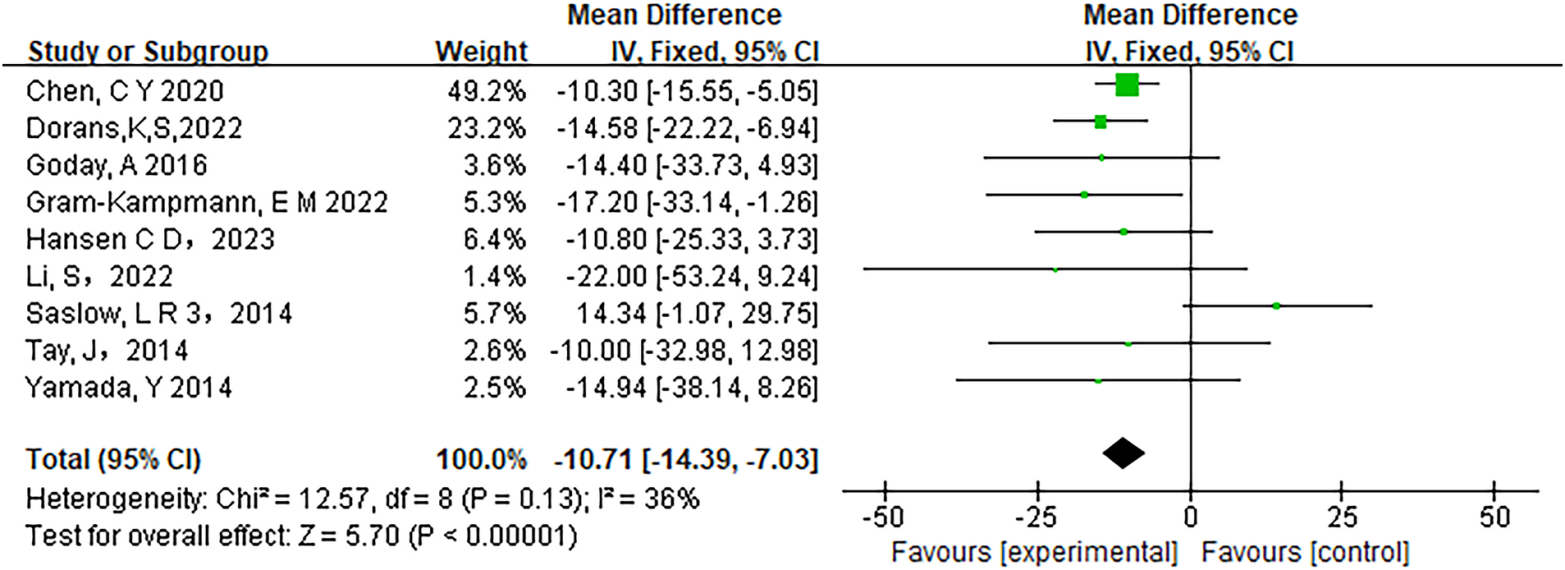
Effect of a carbohydrate diet on FPG.

Fifteen studies assessed the relationship between carbohydrate intake and HbA1c levels. The heterogeneity analysis revealed an I^2^ value of 0% with a *p*-value of less than 0.0001, indicating no observed heterogeneity among the studies. Therefore, a fixed-effects model was appropriate for the analysis. The pooled results showed a significant reduction in HbA1c levels in participants adhering to low-carbohydrate diets compared to those on control diets (mean difference [MD] = −0.36, 95% CI −0.44 to −0.29, *p* < 0.00001) (Figure 3). Similarly, Fasting Plasma Glucose (FPG) levels improved significantly in the low-carbohydrate diet group across 10 studies (MD = −10.71, 95% CI -14.39 to −7.03, *p* < 0.00001) (Figure 4).

#### 3.3.2 Effect of a carbohydrate diet on lipid profiles

**Figure 5:**
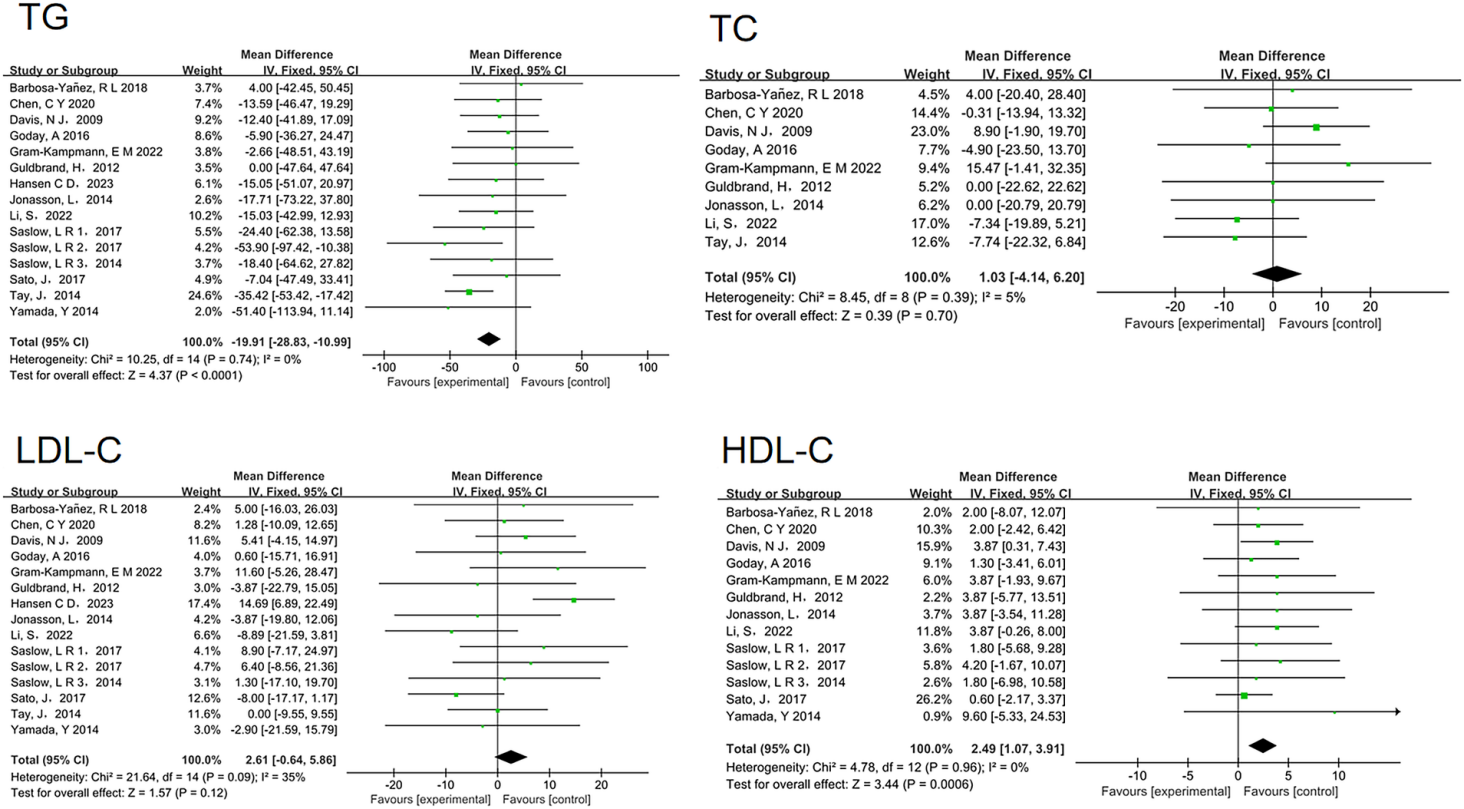
Effect of a low carbohydrate diet on triglycerides, high-density lipoprotein cholesterol, low-density lipoprotein cholesterol, total cholesterol.

Furthermore, the analysis revealed beneficial changes in lipid profiles associated with low-carbohydrate diets. There was a significant reduction in triglyceride levels (MD = −19.91, 95% CI [−28.83, −10.99], *p* < 0.00001) and a significant increase in HDL-C levels (MD = 2.49, 95% CI [1.07, 3.91], *p* = 0.0006). However, no significant differences were observed in LDL-C levels (MD = 2.61, 95% CI [−0.64, 5.86], *p* = 0.12) or TC levels (MD = 1.03, 95% CI [−4.14, 6.20], *p* = 0.7) (Figure 5).

### 3.4 Other metabolic outcomes

**Figure 6:**
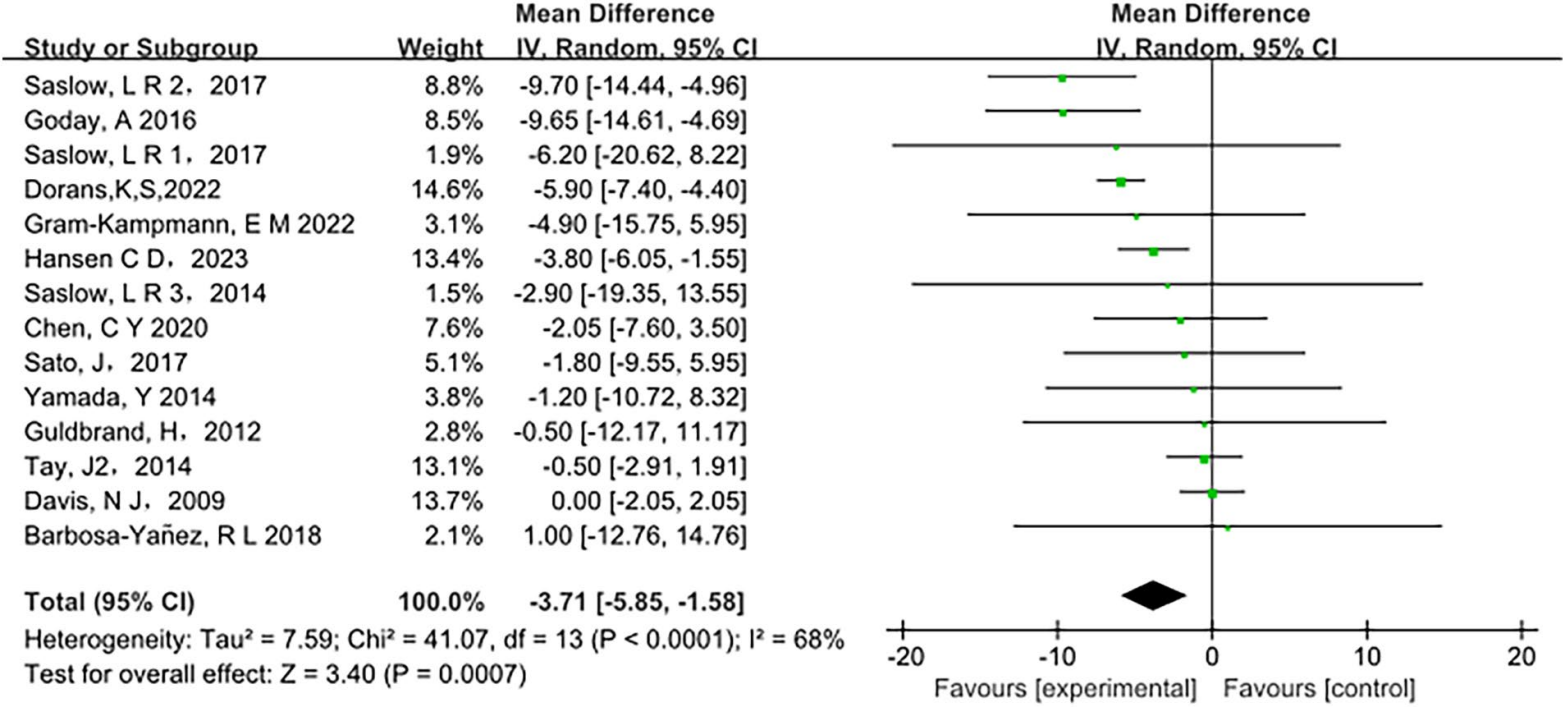
Effect of a carbohydrate diet on weight.

**Figure 7:**
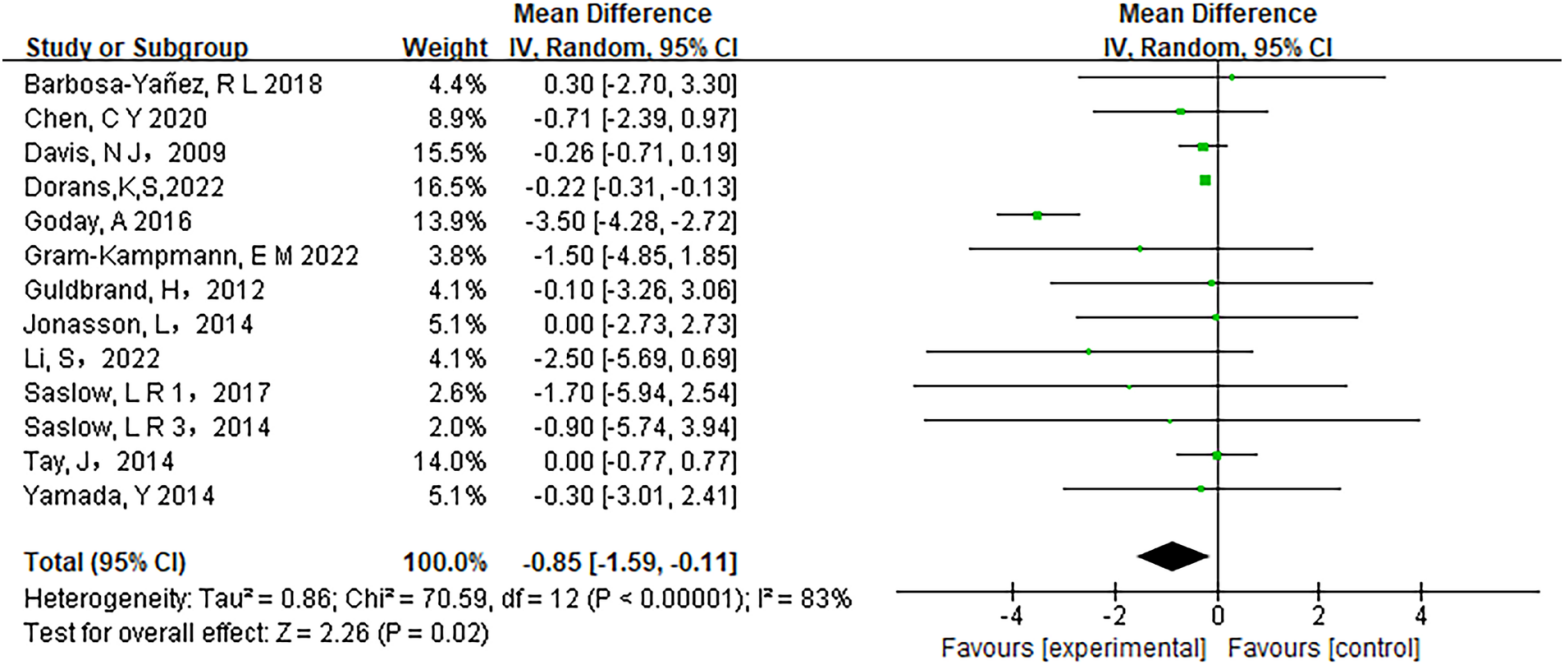
Effect of a carbohydrate diet on BMI.

**Figure 8:**
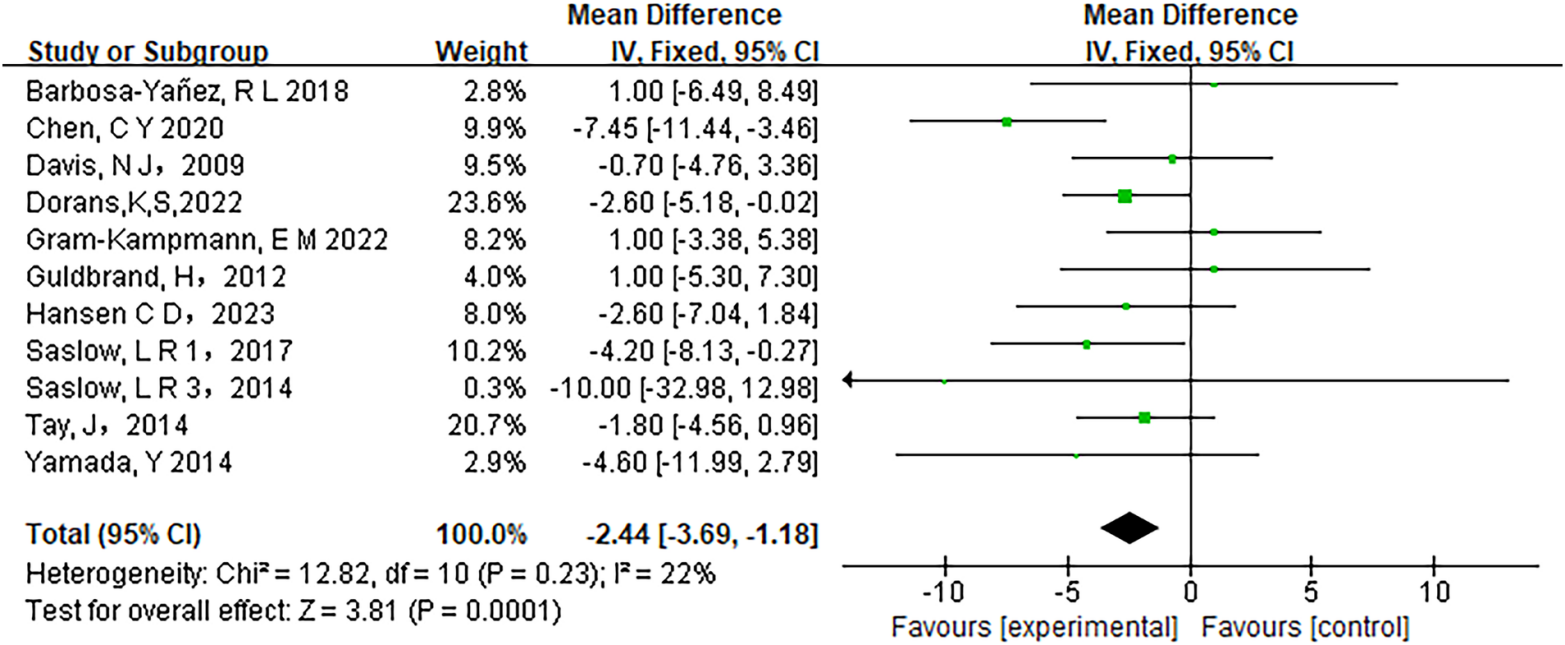
Effect of a carbohydrate diet on DBP.

**Figure 9:**
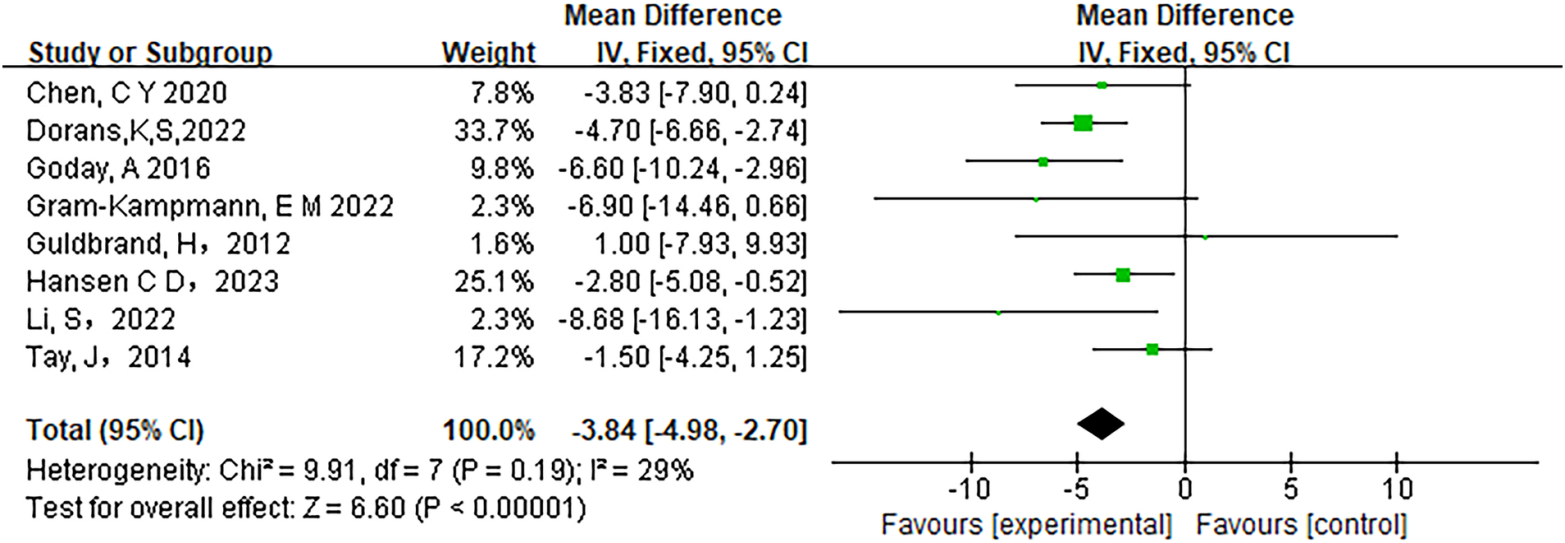
Effect of a carbohydrate diet on waist circumference.

**Figure 10:**
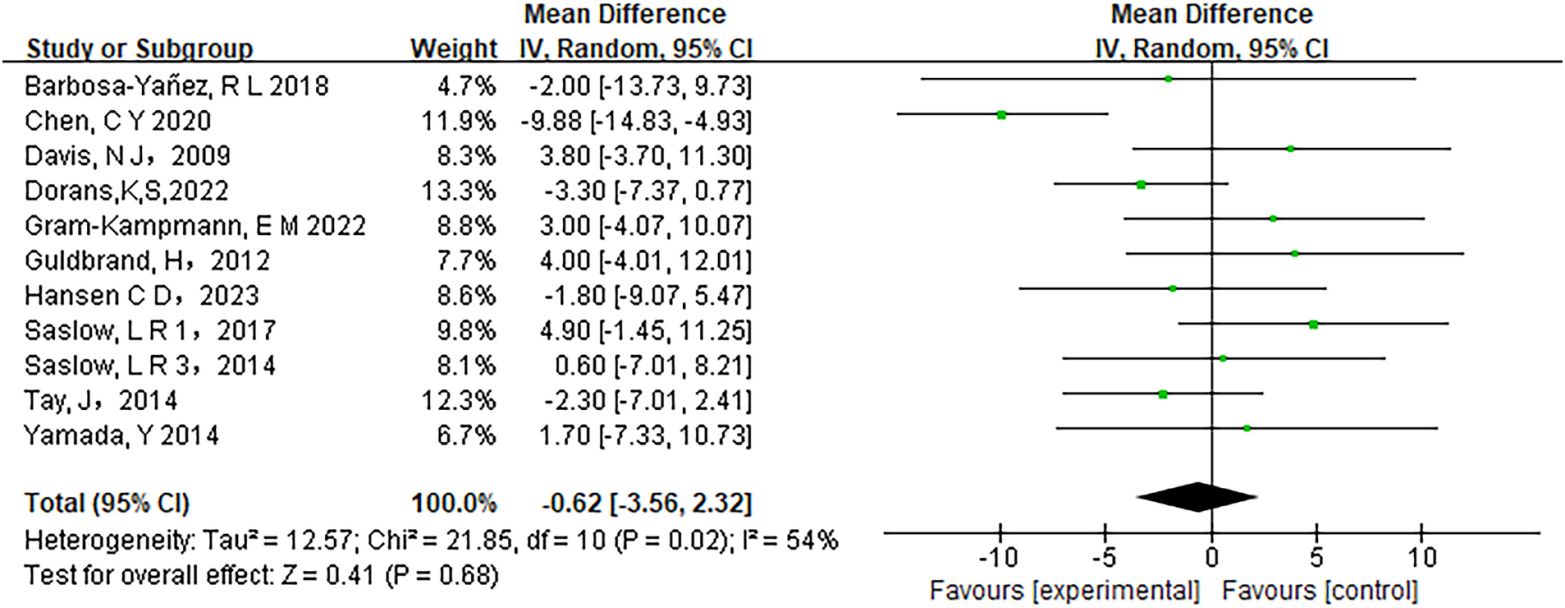
Effect of a carbohydrate diet on SBP.

Low-carbohydrate diets also resulted in substantial reductions in several key health metrics. Participants following these diets experienced significant decreases in body weight (MD = −3.71, 95% CI [−5.85, −1.58], *p* < 0.05) (Figure 6), body mass index (BMI) (MD = −0.85, 95% CI [−1.59, −0.11], *p* < 0.05) (Figure 7), diastolic blood pressure (DBP) (MD = −2.44, 95% CI [−3.69, −1.18], *p* < 0.05) (Figure 8), and waist circumference (MD = −3.84, 95% CI [−4.98, −2.70], *p* < 0.05) (Figure 9). Each of these parameters showed significant improvements in the low-carbohydrate diet group, highlighting the diet’s potential to enhance various aspects of metabolic health. However, no significant changes were observed in systolic blood pressure (SBP) (MD = −0.62, 95% CI [−3.56, 2.32], *p* = 0.62) (Figure 10).

### 3.5 Rare side effects

Among the included articles, 12 studies addressed adverse reactions. Of these, two studies reported no observed adverse reactions, while three did not explicitly mention adverse reactions. The types of mild adverse events (AEs) reported included asthenia, headache, nausea, and vomiting. These symptoms were most prevalent during the first 2 weeks (15 days) of the studies and tended to diminish over time. Notably, only one participant withdrew from the study due to nausea linked to the ketogenic diet.

There was a higher incidence of adverse events related to hypoglycemia, with 14 instances of hypoglycemic symptoms and seven documented hypoglycemic events. Although more participants reported experiencing hypoglycemic episodes, no severe hypoglycemic events were recorded. Among these cases, six patients who were receiving treatment with sulfonylureas or insulin experienced symptomatic hypoglycemia. One participant in the control group, potentially due to very low-calorie intake, and another in the intervention group, who was taking metformin, also experienced hypoglycemic symptoms. However, these adverse events did not recur once the medication dosages were adjusted. Importantly, no patients exhibited ketonuria throughout the study period. In terms of other adverse events, six cases of constipation, two cases of diarrhea, and four cases of abdominal discomfort were clearly documented. Aside from these, there were no significant differences in other self-reported potential adverse events between the low-carbohydrate diet group and the control group.

In the low-carbohydrate diet group, serum uric acid levels demonstrated an increasing trend post-intervention, potentially elevating the risk of gout attacks. During the study, 11 participants (five from the experimental group and six from the control group) encountered musculoskeletal issues while undergoing exercise training. These participants were able to continue in the study after their recovery.

Significant adverse events included one participant in the low-carbohydrate diet group being diagnosed with prostate cancer. Three participants in the high-carbohydrate group underwent elective surgeries. Additionally, four participants (three from the low-carbohydrate diet group and one from the high-carbohydrate group) experienced work-related injuries that were unrelated to the study. One participant in the high-carbohydrate diet group was involved in a motor vehicle accident.

A severe adverse event occurred in the low-carbohydrate, high-fat (LCHF) diet group, where one participant developed severe hypertriglyceridemia, with triglyceride levels reaching 12 mmol/L (1,062 mg/dL). This participant withdrew from the study but saw their triglyceride levels normalize following treatment with gemfibrozil.

Lastly, a cardiac event was reported 3 weeks into the study. However, the Data and Safety Monitoring Board determined that this event was unrelated to the dietary intervention.

### 3.6 Heterogeneity and publication bias

Analysis of heterogeneity revealed I^2^ values of 68% for the weight primary outcomes, indicating high heterogeneity. This study (52) have some medication adjustment: During the study period, adjustments were made to diabetes medications to reduce side effects that could potentially affect the study outcomes. This adjustment may have had an impact on weight changes (49). We considered several factors when analyzing the potential reasons for heterogeneity in weight changes: 1. Content of dietary intervention: The low saturated fat diet and the low-fat diet in the literature have similarities in the distribution of macronutrients, which could lead to different effects on weight loss. Although both diets were low-calorie, the specific levels of energy intake might differ, which could affect the extent of weight loss. 2. The literature mentioned that participants engaged in structured exercise during the study period, which could contribute to weight loss. Variations in the levels of physical activity across different studies might also influence the outcomes of weight loss. 3. The literature used a mixed model ANOVA and the Johnson-Neyman procedure to analyze the data, which might differ from the statistical methods used in other studies. Different statistical methods might yield different results.

We considered several factors when analyzing the potential reasons for heterogeneity in BMI changes: 1. Study Design: The study was a prospective, open-label, multicenter randomized clinical trial, which may differ in design from other studies. Differences in study design could lead to heterogeneity in the results. 2. Intervention: In addition to dietary intervention, this study also included lifestyle and behavioral change support (Diaprokal Method). This comprehensive intervention might yield different results compared to studies that focus solely on diet or solely on behavioral changes. 3. Comorbidities and Medication Use: The comorbid conditions and diabetes medications of the participants may affect BMI. For instance, the use of insulin or other medications that impact weight could alter the baseline level of BMI and its changes over time.

## 4 Discussion

In our study, we performed a meta-analysis to evaluate the effects of low-carbohydrate diets on glucose and lipid metabolism in overweight or obese individuals with T2DM. Glycemic control, a key indicator of blood glucose management, was assessed through measurements of HbA1c and FPG levels. The meta-analysis demonstrated a significant reduction in both HbA1c and FPG levels for individuals following low-carbohydrate diets compared to those on control diets. These findings provide valuable insights for optimizing dietary management strategies for patients with diabetes.

Additionally, we analyzed blood lipid parameters, including TC, LDL-C, HDL-C, and TG. When comparing the low-carbohydrate diet group to the control group, we observed significant increases in HDL-C levels and significant decreases in TG levels within the low-carbohydrate diet group. However, no significant differences were noted in TC and LDL-C levels between the two groups. The selective improvement in HDL-C and TG levels, without significant changes in LDL-C and TC, can be attributed to several physiological and biochemical mechanisms influenced by low-carbohydrate diets.

Low-carbohydrate diets typically increase the reliance on fat as a primary energy source, which enhances lipolysis and fat oxidation. This shift in metabolism can lead to a reduction in TG levels, likely due to decreased synthesis of very-low-density lipoprotein, a precursor to triglyceride-rich lipoproteins (55). Additionally, increased intake of fats, particularly monounsaturated and polyunsaturated fats, can promote the synthesis of HDL particles, thereby elevating HDL-C levels (56). Reduced carbohydrate intake leads to lower insulin levels, which plays a key role in lipid metabolism. Lower insulin levels decrease the inhibition of hormone-sensitive lipase, promoting the breakdown of triglycerides and the release of free fatty acids from adipose tissue (57, 58). Improved insulin sensitivity can also enhance HDL-C production and function, contributing to the elevation of HDL-C levels (59, 60).

Changes in lipoprotein particle distribution may also contribute to the observed lipid profile changes. Studies have shown that low-carbohydrate diets can increase the size of LDL particles, shifting them from small, dense LDL (which are more atherogenic) to larger, more buoyant LDL particles (61, 62). This shift may not significantly alter overall LDL-C concentrations, but it could improve the lipid profile by reducing the proportion of harmful LDL subtypes. Furthermore, weight loss, a common outcome of low-carbohydrate diets, independently contributes to improvements in lipid profiles. A reduction in adipose tissue mass decreases the secretion of pro-inflammatory cytokines and improves lipid metabolism, leading to elevated HDL-C and reduced TG levels (63, 64). However, the effects on LDL-C and TC may vary depending on individual metabolic responses and the types of fats consumed in the diet.

The quality and types of fats consumed in low-carbohydrate diets can influence lipid outcomes. Diets rich in saturated fats may have different effects on LDL-C compared to those high in unsaturated fats (65, 66). In our analysis, the lack of significant changes in LDL-C and TC could be attributed to variability in fat quality across the included studies. Diets emphasizing unsaturated fats may enhance HDL-C without adversely affecting LDL-C levels, thereby maintaining overall TC within a stable range (67, 68).

Regarding other metabolic outcomes, low-carbohydrate diets were associated with significant reductions in body weight, BMI, DBP, and waist circumference compared to control diets. These findings underscore the potential of low-carbohydrate diets to improve a wide range of metabolic health indicators in patients with T2DM.

In the short term (e.g., 8 weeks), low-carbohydrate diets have been consistently shown to produce rapid improvements in anthropometric parameters such as BMI and body weight (69, 70). This swift weight loss is primarily due to initial water loss and glycogen depletion, as glycogen stores are depleted with reduced carbohydrate intake and are later replenished when carbohydrates are reintroduced (70). Additionally, the higher protein and fat content of low-carbohydrate diets can enhance satiety, leading to a natural reduction in caloric intake without the need for conscious calorie counting (71, 72). These factors collectively contribute to the accelerated weight loss observed in the early stages of low-carbohydrate dietary interventions.

However, evidence from longer-term studies (e.g., 6 months to 1 year) suggests that the distinctive advantages of low-carbohydrate diets in reducing body weight and BMI may attenuate over time, with outcomes converging toward those of balanced low-calorie diets (73–76). Several factors contribute to this phenomenon: Over extended periods, the body may undergo metabolic adaptation, where basal metabolic rates adjust in response to sustained caloric intake and changes in macronutrient composition, potentially slowing further weight loss or leading to weight regain (77, 78). Additionally, maintaining strict carbohydrate restrictions can be difficult for many individuals, often resulting in decreased adherence over time. Adherence issues may arise from dietary fatigue, social limitations, or the restrictive nature of the diet, all of which can hinder long-term success. A recent study emphasized that although low-carbohydrate diets can yield significant short-term improvements, maintaining these dietary changes over extended periods is challenging without continuous support and motivation (79). Reduced compliance diminishes the efficacy of low-carbohydrate diets, as their benefits are closely linked to sustained carbohydrate limitation.

In contrast, balanced low-calorie diets may be easier to adhere to over the long term because they allow for greater flexibility in macronutrient intake. This flexibility can enhance adherence and sustainability, potentially resulting in weight loss outcomes comparable to those of low-carbohydrate diets. Long-term dietary changes also require significant behavioral adjustments. The restrictive nature of low-carbohydrate diets can affect psychological well-being, leading to decreased motivation and a higher likelihood of reverting to previous eating habits, which can impact long-term weight management (80).

These findings underscore the importance of considering both short-term and long-term outcomes when recommending dietary interventions for overweight or obese patients with T2DM. While low-carbohydrate diets may offer rapid initial benefits, the long-term efficacy and sustainability of these diets compared to more balanced approaches need further investigation. Therefore, personalized dietary plans that take into account patient preferences, lifestyle, and the likelihood of sustained adherence may be more effective in achieving lasting weight management and metabolic health. In conclusion, while low-carbohydrate diets can lead to rapid improvements in anthropometric parameters and some cardiometabolic markers, their long-term efficacy compared to balanced low-calorie diets remains unclear. A more flexible and individualized dietary approach may promote better adherence and overall health, making it a promising strategy for long-term weight management and metabolic health.

In addition to the population of overweight or obese individuals with T2DM evaluated in our meta-analysis, the effects of low-carbohydrate diets have been examined in diverse cohorts, offering further insights into their broader applicability. For instance, a meta-analysis of randomized controlled trials conducted in generally healthy overweight adults found that low-carbohydrate diets resulted in greater short-term weight loss compared to low-fat diets, reinforcing the potential utility of carbohydrate restriction in weight management beyond the diabetic population (81). Similarly, studies conducted in individuals with metabolic syndrome have shown that low-carbohydrate eating patterns can improve insulin sensitivity, triglyceride levels, and HDL-C concentrations, underscoring their potential to mitigate multiple cardiometabolic risk factors in populations at heightened cardiovascular risk (11).

Research has also explored the impact of low-carbohydrate diets in ethnic populations and those with varying baseline dietary patterns. For example, investigations in Asian populations, who typically consume diets high in refined carbohydrates, have reported significant reductions in HbA1c and body weight among individuals with T2DM following carbohydrate-restricted interventions (82). Additionally, Mediterranean populations, often consuming moderate-carbohydrate diets enriched in unsaturated fats, have demonstrated improvements in TG and HDL-C when carbohydrate intake is selectively reduced, suggesting that even moderate carbohydrate reductions within culturally familiar dietary frameworks can yield metabolic benefits (83, 84).

Furthermore, the favorable metabolic effects of low-carbohydrate diets have been documented in conditions such as polycystic ovary syndrome, where improvements in insulin resistance, body weight, and lipid profiles have been observed (85). This indicates that the underlying mechanisms—reduced insulin levels, enhanced lipolysis, and improved lipoprotein particle profiles—may extend to metabolic disturbances beyond T2DM, making low-carbohydrate dietary interventions relevant for a variety of endocrine and metabolic disorders.

Our findings align with those of previous studies, corroborating the effectiveness of low-carbohydrate diets in managing metabolic health. The increasing popularity of these diets can be attributed to their demonstrated efficacy in promoting weight loss and improving glucolipid metabolism (86–89). Low-carbohydrate diets are associated with greater short-term weight loss compared to diets that do not restrict carbohydrates. Additionally, they have a favorable long-term impact on cardiovascular risk factors (75). Compared to low-fat diets, low-carbohydrate diets resulted in significantly greater reductions in TG (−0.14 mmol/L; 95% CI, −0.18 to −0.10 mmol/L), DBP (−0.87 mmHg; 95% CI, −1.41 to −0.32 mmHg), and body weight (−1.33 kg; 95% CI, −1.79 to −0.87 kg). Additionally, low-carbohydrate diets led to a significant increase in HDL cholesterol (0.07 mmol/L; 95% CI, 0.06 to 0.09 mmol/L). These results were observed over a period of 6 to 23 months, based on a meta-analysis of 3,939 participants (90). Another meta-analysis involving 6,499 adults concluded that low-carbohydrate diets are effective for weight loss and for improving certain aspects of the lipid profile, including increases in HDL cholesterol and reductions in TG. However, these benefits are accompanied by an increase in LDL and TC levels (91).

It is worth noting that a consensus statement highlights that low-carbohydrate diets can significantly enhance glycemic control and promote weight loss in adults with T2DM (92). Goldenberg et al. (93) conducted a meta-analysis which found that low-carbohydrate diets significantly increased the rate of diabetes remission compared to control diets (57% vs. 31%), with remission defined as achieving an HbA1c level below 6.5%. Additionally, their analysis highlighted significant improvements in weight loss, TG, and insulin sensitivity in those adhering to low-carbohydrate diets. These benefits were pronounced at the six-month mark but diminished in magnitude by the 12-month evaluation. These findings align with another meta-analysis, which suggests that low-carbohydrate diets may offer minor short-term improvements in HbA1c levels and body weight. However, these benefits do not appear to be sustained over the long term (22). Furthermore, a meta-analysis produced results that diverged from those of the previously mentioned studies. This analysis indicated that reductions in HbA1c and triglyceride levels were slightly more pronounced with low-carbohydrate diets compared to high-carbohydrate diets. However, it found no significant differences between the two dietary groups concerning weight, HDL and LDL cholesterol levels, TC, or blood pressure (21).

Our study focuses specifically on overweight or obese patients with T2DM, a more distinctive group compared to previous studies that typically addressed either obesity/overweight or diabetes alone. This targeted approach allows us to highlight the significant effects of low-carbohydrate diets on this particular population, providing tailored dietary plan options that meet their unique needs. Furthermore, our study encompasses a broader range of control diets, including low-fat, high-carbohydrate, and standard diabetic diets, thereby offering a more comprehensive comparison than previous studies.

This study has several limitations. The included trials showed variability in the specifics of dietary interventions, participant adherence, and study duration, which contributed to some heterogeneity in the outcomes. Additionally, the reliance on self-reported dietary intake in several studies could introduce bias. Future research should aim to standardize dietary interventions, enhance the use of objective adherence measures, and explore the long-term sustainability and health impacts of low-carbohydrate diets. Further investigation into the mechanistic underpinnings of these diets’ effects on glucose and lipid metabolism could provide valuable insights for optimizing dietary recommendations for the management of T2DM and obesity.

The results of this meta-analysis have significant implications for clinical practice. Given the observed improvements in critical metabolic markers, low-carbohydrate diets should be considered a viable intervention for managing T2DM. This dietary approach could be particularly advantageous for patients who have had difficulty achieving glycemic control or managing their weight with traditional dietary recommendations. However, it is essential to tailor dietary advice to individual patient needs, preferences, and medical conditions, underscoring the importance of a personalized nutrition strategy in clinical practice.

One primary concern with low-carbohydrate diet is the potential for side effects. However, the included studies that reported side effects associated with low-carbohydrate diets generally found no significant common adverse effects. The most frequently noted issues were dehydration-related disorders, transient hypoglycemia, halitosis, gastrointestinal disturbances, hyperuricemia, and changes in the lipid profile. These side effects were typically mild and often resolved spontaneously.

Although the majority of participants were able to complete the studies, a minority did experience health issues during the study period. These adverse reactions could be linked to dietary changes, increased physical activity, or other unspecified factors. It is important to note that these studies did not always explicitly establish a direct causal relationship between the adverse events and the dietary interventions. Nonetheless, these events were documented during the study period.

Overall, the study results suggest that, while some adverse reactions were observed, they were generally mild and tended to decrease over time without leading to serious health issues. However, adherence to a low-carbohydrate diet over the long term appears to be weaker compared to other diets, and dietary compliance often decreases over time. This presents practical challenges in the application of low-carbohydrate diets. Therefore, it is recommended that patients considering a ketogenic or low-carbohydrate diet do so under the supervision of professional medical personnel and that they regularly monitor relevant health indicators.

## 5 Conclusion

After a thorough meta-analysis of the current evidence and taking into account the potential side effects, low-carbohydrate diets appear to significantly improve glycemic control, TG, HDL-C, weight, BMI, DBP, and waist circumference in overweight or obese patients with T2DM. This study provides valuable dietary guidance for managing T2DM in this patient population. Notably, the significant reduction in waist circumference underscores the effectiveness of low-carbohydrate diets in addressing central obesity, a critical factor in metabolic health and cardiovascular risk. These substantial improvements in both anthropometric and metabolic parameters highlight the potential of low-carbohydrate diets as a comprehensive strategy for managing T2DM. Therefore, adopting low-carbohydrate diets could offer multifaceted benefits in controlling metabolic health and reducing obesity-related risks in individuals with T2DM.

## Data Availability

The original contributions presented in the study are included in the article/supplementary material, further inquiries can be directed to the corresponding authors.
